# A Case of ProGlide Knot Entrapment by the Inguinal Ligament Resulting in Hemostasis Failure

**DOI:** 10.1002/ccr3.71139

**Published:** 2025-10-03

**Authors:** Kotaro Mukasa, Yasunori Yakita, Ryosuke Marushima, Shinichiro Abe, Soichi Asano

**Affiliations:** ^1^ Department of Cardiovascular Surgery Chiba Cardiovascular Center Ichihara Japan

**Keywords:** endovascular aneurysm repair, femoral artery, hemostasis failure, inguinal ligament, percutaneous closure device, ProGlide

## Abstract

Entrapment of ProGlide sutures by the inguinal ligament can result in closure failure. Confirming puncture site location under fluoroscopy and careful dissection may help prevent this complication.

## Graphical abstract

Entrapment of ProGlide knots by the inguinal ligament resulting in hemostasis failure after endovascular iliac aneurysm repair.

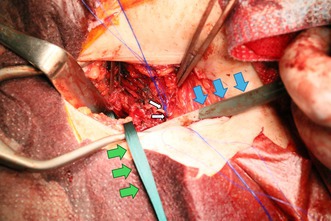

## 1 Case

**FIGURE 1:**
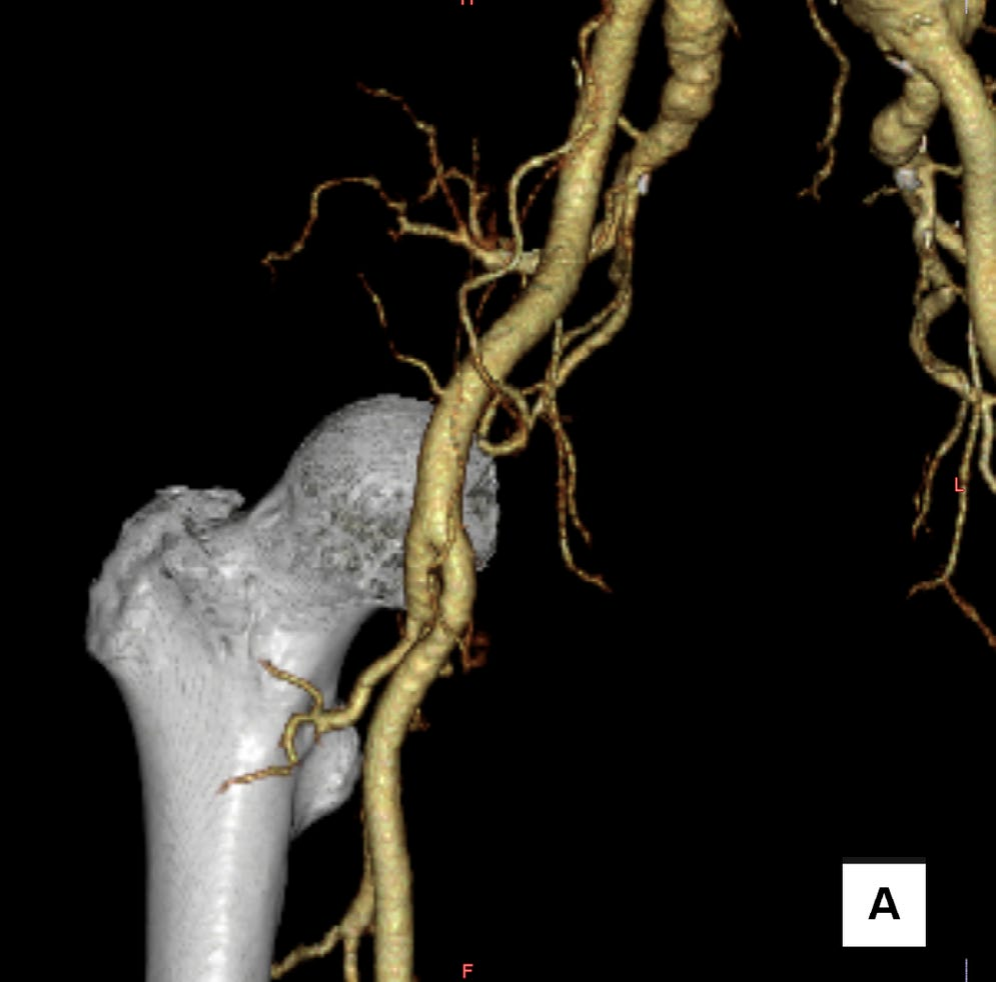
A three‐dimensional reconstruction image of the preoperative contrast‐enhanced computed tomography scan near the puncture site of the right femoral artery. The letter A denotes that the three‐dimensional reconstruction is shown from an anterior frontal view.

A 77‐year‐old male presented with a 47 mm left common iliac artery aneurysm requiring endovascular repair. Preoperative imaging demonstrated that the femoral artery bifurcation was caudal to the inguinal ligament, corresponding to the lower half of the femoral head (Figure 1). The common femoral artery was approximately 9 mm in diameter with minimal anterior calcification. Given favorable vascular anatomy, we elected to use percutaneous access with the ProGlide closure device (Abbott Vascular, Santa Clara, California) utilizing the preclose technique. There was no high femoral artery bifurcation, history of prior groin surgery, or obesity.

Prior to the main procedure, under ultrasound guidance, right common femoral artery access was obtained and two ProGlide sutures were deployed per protocol. We pulled the sutures to check for hemostasis, but neither suture achieved complete bleeding control. However, the bleeding had slowed, so we assumed that hemostasis could be achieved with manual compression after tightening the knots.

**FIGURE 2:**
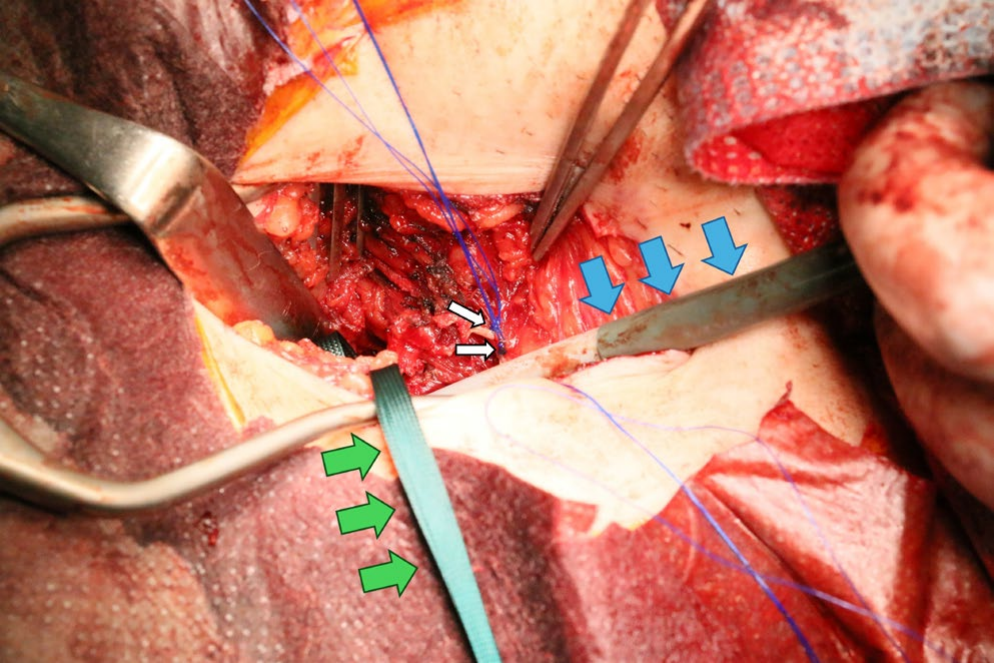
Intraoperative photograph of the right groin incision showing the ProGlide suture knot entrapped by the inguinal ligament. Blue arrow: Sheath inserted into the femoral artery. Green arrow: Tape placed around the femoral artery. The artery itself is obscured and not visible. White arrow: ProGlide suture knot.

Following successful completion of the planned procedure and sheath removal, neither ProGlide suture achieved adequate hemostasis despite attempts at knot tightening. This necessitated conversion to open surgical exposure through a 7 cm vertical groin incision. Surgical exploration revealed that both knots were entrapped by the inguinal ligament, preventing proper apposition to the arterial wall (Figure 2). Hemostasis was ultimately achieved using a purse‐string suture on the common femoral artery.

ProGlide is contraindicated for punctures above the inguinal ligament due to the risks of retroperitoneal hemorrhage and inferior epigastric artery injury. In this case, despite preoperative imaging suggesting appropriate anatomy, the puncture inadvertently traversed the inguinal ligament, resulting in suture entrapment and closure failure. Based on this experience, we have modified our technique in two ways: first, performing meticulous subcutaneous dissection with mosquito forceps to prevent tissue entrapment, and second, routinely confirming the puncture site location under fluoroscopic guidance to avoid high punctures above the femoral head [1]. ProGlide has been reported to require additional surgical repair in 1.7% of cases, highlighting the need for careful use [2]. The patient provided written informed consent for publication of this case report.

## Author Contributions

**Kotaro Mukasa:** conceptualization, data curation, investigation, methodology, writing – original draft, writing – review and editing. **Ryosuke Marushima:** data curation. **Yasunori Yakita:** supervision. **Shinichiro Abe:** supervision. **Soichi Asano:** supervision.

## Ethics Statement

The authors have nothing to report.

## Consent

Written informed consent was obtained from the patient for the publication of this case report and the accompanying images.

## Conflicts of Interest

The authors declare no conflicts of interest.

## Data Availability

Additional data supporting the findings of this study is available from the corresponding author upon reasonable request.
